# 28.4 FLEXIBLE BODY BOUNDARY AND ALTERED MAPPING OF THE BODILY SELF IN THE SCHIZOPHRENIA SPECTRUM: CAUSES, PROCESSES AND POTENTIAL INTERVENTION

**DOI:** 10.1093/schbul/sby014.119

**Published:** 2018-04-01

**Authors:** Sohee Park, Lénie Torregrossa, Taylor Benson, Lauri Nummenmaa, Matthew Snodgress, Enrico Glerean, Eon Sol Chon, Seok Jin Hong

**Affiliations:** 1 Vanderbilt University; 2 University of Turku

## Abstract

**Background:**

Our sense of embodied self depends on continuous spatiotemporal integration and predictive coding of multisensory signals to yield a stable internal landscape. However, schizophrenia is characterized by inconsistent mapping of the physical and parasomatic body space, autoscopic hallucinations and flexible body self boundary. We aimed to elucidate the specific roles of exteroceptive, proprioceptive and interoceptive systems in generating self disturbances. Lastly, if schizophrenia represents one end of the spectrum of bodily self disorders, it is also important to understand what lies at the other extreme end, represented by those whose prediction coding is honed to perfection from years of training (athletes) to gain insight into potential remediation strategies.

**Methods:**

In Study 1, components of bodily self-disturbances were examined in individuals with schizophrenia (SZ), matched controls (CO) and prodromal participants (P) with tasks that assessed tactile perception (2-point discrimination task), susceptibility to proprioceptive-tactile illusions, multisensory integration, visual body mapping of emotions (emBODY), and interoceptive awareness (heartbeat detection task). Phenomenological dissociative experiences were captured with a novel picture-based inventory (BODI). In Study 2, we recruited healthy participants with extraordinary expertise to coordinate interoceptive, proprioceptive and exteroceptive signals to perform physical tasks (athletes), and compared their embodiment of emotions with that of matched controls and individuals with schizophrenia.

**Results:**

Individuals with schizophrenia and prodromal participants were impaired in interoceptive awareness, exteroceptive tactile discrimination, and audio-visual integration compared with matched control groups. SZ and P also showed increased sensitivity to proprioceptive illusions, which was associated with increased dissociative experiences and positive syndromes. Bodily sensations associated with emotions were reduced in SZ and P compared to CO. Importantly, the spatial locations of embodied emotions were different in SZ compared with CO. Interestingly, athletes showed highly precise localization of embodied emotions compared with matched controls. Self-disturbances were exacerbated by social isolation regardless of diagnosis.

**Discussion:**

These results suggest that mapping of internal signals to the experience of external world is inconsistent or incoherent, contributing to fragmented and discontinuous self experience in persons with schizophrenia. More specifically, proprioceptive prediction errors seem to contribute to abnormally flexible self boundary. Diminished access to interoceptive signals may lead to reduced mapping of bodily sensations. Embodied emotions were reduced in SZ and P compared to CO. Athletes seemed to have much more precisely tuned awareness of embodied emotions. These results are consistent with the framework of increased internal neural noise in schizophrenia, which could lead to both weakened and poorly integrated interoceptive, proprioceptive and exteroceptive signaling, and a fragmented sense of self. Athletes data suggest that it may be possible to remediate bodily self disturbances via physical training. These findings underscore the importance of bringing back the body to psychiatry.

